# Designing and validating a comparison card method for quantification of glenoid bone defect

**DOI:** 10.1038/s41598-022-20908-y

**Published:** 2022-10-06

**Authors:** Liang Chen, Yichong Zhang, Yufeng Wu, Jingyang Chen, Zexin Hong, Jiabao Ju, Jianhai Chen, Dawei Gao

**Affiliations:** 1 Traditional Chinese Medicine Hospital of Zhongshan, Guangzhou University of Chinese Medicine, 3 Kangxin Road, Zhongshan, 528401 Guangdong China; 2 Department of Trauma & Orthopedics, Peking University People’s Hospital, 11 South Xizhimen Street, Beijing, 100044 China

**Keywords:** Diagnosis, Medical imaging, Trauma

## Abstract

To design and investigate a comparison card to evaluate the glenoid bone defect compared with Sugaya method. 33 patients with bony Bankart lesions were included. The comparison card and Sugaya method were performed on two occasions by three participants. The intra-group correlation coefficient (ICC) analysis and the inter-group correlation coefficient analysis of two measurements was performed. The concordance of the two methods was assessed using Bland–Altman analysis. Firstly, the percentage of defect measured by Sugaya method was 10.32 ± 8.38, and the comparison card method was 10.26 ± 8.41, 10.15 ± 8.23, and 10.62 ± 8.48, separately. There was no statistically significant difference (*P* > 0.05). The second measurement showed it was 10.37 ± 8.39 for Sugaya method, and 10.23 ± 8.37, 10.15 ± 8.35, 10.54 ± 8.49 for the comparison card, without a statistically significant difference (*P* > 0.05). For the comparison card, the intra- and inter-observer ICC values were all > 0.75. In the first measurement, Bland–Altman analysis demonstrated agreement between the two methods (bias, −0.03; SD, 0.48; − 0.97– 0.91; 95% CI, − 0.1999– 0.1413). Agreement was also found between them (bias, 0.07; SD, 0.61; − 1.13– 1.26; 95% CI, − 0.1509– 0.2812) in the second measurement. The comparison card method has similar accuracy with Sugaya method, which is of great reliability and convenience.

## Introduction

Fractures of the anteroinferior aspect of the glenoid rim, known as bony Bankart lesions, can occur frequently in the setting of traumatic anterior shoulder dislocation, causing anterior instability of shoulder^1,2^. An osseous Bankart lesion is usually secondary to direct violence or recurrent erosion of shoulder dislocation, manifested as compression or bone defect, mainly located at the anterior rim of glenoid. Glenoid bone loss can be seen in about 40% of patients with first discovered dislocation and 85% of patients with recurrent dislocation^3^. Previous studies have demonstrated that the bone loss was associated with great risk of re-dislocation after surgical repair^4^. Therefore, it is crucial to evaluate the glenoid bone loss threshold value for therapeutic decision-making, and accurate preoperative quantification is paramount in preventing surgical failure ^5,6^.

The evaluation of glenoid defect is of controversy. Several methods have been described to measure the amount of glenoid bone defect, including radiogram, Computed Tomography (CT) scan, Magnetic Resonance Imaging (MRI), and arthroscopy^7^. But in determining surgical procedure, preoperative 3D CT could provide the most accurate, reliable, and reproducible estimation of glenoid bone loss^6^. Sugaya method is widely used and highly recognized based on the circle formed by inferior glenoid^8^. However, the usage is limited in some circumstances due to the complicated software calculation. Also it’s hard to draw an exact curve if the defect shape was irregular. Nevertheless, it is increasingly necessary to develop a new method to detect and measure the glenoid defect with convenience and accuracy. The purpose of our study is to design a comparison card to evaluate the glenoid bone defect quickly and accurately, and validate its intra-observer and inter-observer reliability at the same time.

## Results

A total of 33 patients with bony Bankart lesions were included in the study, including 26 males and seven females from August 2016 to May 2020, with an average of 37.5 years of age.

All patients were measured by the two methods. The first results from different observers had no statistically significant difference (*P* > 0.05). The second results after three months were not statistically different among observers as well (*P* > 0.05) (Table 1). The intra- and inter-observer ICC values were all > 0.75, and the reliability was good (Table 2).

**Table 1:** Percentage of glenoid bone defect measured by the Sugaya method and the comparison card method (n = 33), %, mean ± standard.

	Sugaya method	Participant A	Participant B	Participant C	*P*
First	10.32 ± 8.38	10.26 ± 8.41	10.15 ± 8.23	10.62 ± 8.48	0.966
Second	10.37 ± 8.39	10.23 ± 8.37	10.15 ± 8.35	10.54 ± 8.49	0.998

**Table 2:** Inter- and intra-observer ICC analysis (n = 33).

	Inter-observer ICC	Participant A Intra-observer CC	Participant B Intra- observer CC	Participant C Intra- observer CC
First	0.995	0.999	0.999	0.996
Second	0.996			

**Figure 1:**
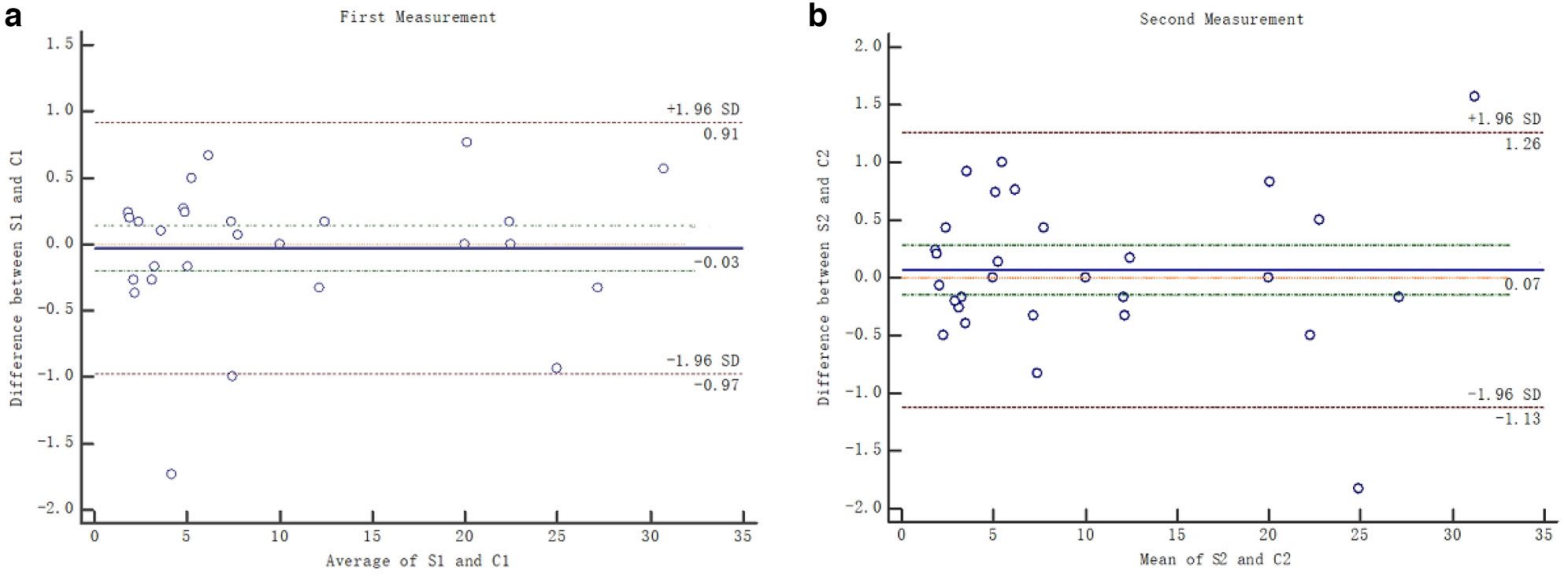
(**a**, **b**) Illustration of the Bland–Altman plot indicating the level of agreement between Sugaya and comparison card measurement of glenoid defect (two measurements).

In the first measurement, concordance was found between Sugaya method and the comparison card (bias, − 0.03; SD, 0.48; lower limit, − 0.97; upper limit, 0.91; 95% CI, − 0.1999−0.1413) using Bland–Altman analysis. In the second measurement, concordance was also found between the two methods (bias, 0.07; SD, 0.61; lower limit, − 1.13; and upper limit, 1.26; 95% CI, − 0.1509−0.2812). It was showed that most plot were within the average area of ± 1.96, showing good equivalence. Figure 1a, b showed the difference in glenoid defect between Sugaya and comparison card method was clinically nonsignificant.

## Discussion

Glenoid rim fractures can be classified into three types: Type I, a displaced avulsion fracture with attached capsule; Type II, a medially displaced fragment malunited to the glenoid rim; and Type III, erosion of the glenoid rim with less than 25% (Type IIIA) or greater than 25% (Type IIIB) deficiency^9^. Any fracture of glenoid rim can decrease the glenohumeral contact area and disrupt the inferior glenohumeral ligament, altering the static stabilizers and predisposing patients to recurrence. Likewise, glenoid bone loss, either from an acute low-energy compression fracture or chronic attenuation secondary to recurrent shoulder dislocations, may also contribute to anterior instability by decreasing the articular arc length and reducing the surface area that resists shear and axial forces^10^. Kim^11^ reported that if the defect was less than 15%, surgical repairment of labrum could restore the stability. But if the defect was greater than 25%, osseous reconstruction was required to obtain better postoperative shoulder function. Therefore, it was critical to precisely quantify the bone loss for making surgical decisions.

The assessment of bone loss, ranging from classic radiographs, through CT with 2D and 3D reconstructions and MRI has been widely described^7^. Radiogram is easy to obtain and the loss of sclerotic glenoid line (LSGL) on anteroposterior radiographs is a moderately sensitive but highly specific finding for anterior glenoid rim defects^12^. However, not being able to quantify the glenoid bone defect has limited the clinical usage. MRI is irreplaceable in evaluating soft tissue and reducing fluorescence exposure, some studies have demonstrated its usage in measuring glenoid bone defects, but not as accurate as three-dimensional CT^13^. Arthroscope is an invasive method, and the position of bare spot is still controversial, which may overestimate the defect size.

Compared with other methods, 3D CT is the golden standard for measuring glenoid defects. The best-circle method is widely used and well recognized, including Barchilon method, Sugaya method, and Pico method^7^. In Barchilon method, the anterior area is calculated as the ratio between the depth and radius of inferior glenoid circle^14^. Because of the irregular defect edges, different measurement points have different distances from the center of circle. Therefore, the measurement error cannot be ignored and the applicationis limited.

The principle and calculation of Sugaya method and Pico method are similar. In Sugaya method^8^ we draw a circle that adequately fit the inferior aspect of glenoid. If there were free bone fragments, their area could be calculated by computer software directly. If there were no bone fragments, the missing part of circle was equivalent to the bone defect, and the proportion of defects equaled the ratio between the missing area and best-fit circle. Similarly, Pico method^15^ is to perform a CT scan of bilateral shoulders to reconstruct the enface view of both glenoids. By drawing a best-fit circle in the contralateral glenoid, we moved this circle to the affected side. The area of missing part is calculated by software to obtain the amount of bone defect and defect ratio.

Magarelli^16^ have demonstrated that Pico method was reliable in measuring glenoid bone defects. However, both Sugaya method and Pico method require the aid of professional software and a certain calculation process, which is difficult for physicians to master and popularize. Due to the limited popularity of computer software and difficulty in software learning, some surgeons made an approximate estimation of defect through visual inspection. But the accuracy is hard to guarantee. Therefore, it is of great essential to develop an method that is easy to use and can measure the glenoid defect accurately at the same time.In this study, we combined digital graphic analysis and Riemann Integral algorithm to design a comparison card (vector diagram) and used the statistical analysis to validate the inter-observer and intra-observer reliability of the comparison card. In addition, we compared the performance of the comparison card method and Sugaya method, which had similar accuracy. Although the design process was a little complicated, we can print the shape and lines representing various amounts of bone loss on transparent film and use it as a template. This will aid surgeons in using zoom feature to correctly size the glenoid to match and read the defect ratio directly in future clinical practice. This design isn’t limited by structure variation and is applicable in different centers. Besides, it can avoid the complicated calculation process and software learning, and be mastered without learning process. Nevertheless, the comparison card makes it possible for physicians in different levels to measure tbone defect precisely and quickly, relieving them from complicated software manipulations.

There are some limitations of this study. First the limited sample size was one of the shortcomings of this study. Also Moroder has found if there were some degrees between observer and en face view of the glenoid, there would be some bias in the measurement of bone loss^17^. In this method, we still need a 3D image of the glenoid to measure, which might bring in some bias.

## Conclusion

The comparison card method has similar accuracy with Sugaya method, which is of great reliability and convenience.

## Methods

### Participants

We included patients with unilateral anterior glenohumeral instability with osseous Bankart lesion, who were admitted to the Third Department of Orthopedics, Zhongshan Hospital of Guangdong Traditional Chinese Medicine University from August 2016 to May 2020. Approval was granted by the Ethics Committee of Traditional Chinese Medicine Hospital of Zhongshan (2021ZXZY-LLK-316). Written informed consent was obtained from patients. All methods were performed in accordance with the relevant guidelines and regulations.

Exclusion criteria: shoulder joint deformity, rheumatism, rheumatoid arthritis, severe osteoarthritis and other diseases that may cause abnormal glenoid morphology.

### CT data

The CT images were taken with a continuous axial 0.625-mm slice thickness. The Digital Imaging and Communications in Medicine (DICOM) data of CT images were exported and analyzed using Mimics software 17.0 (Belgium Materialize Company), to make a multiplanar reconstruction.

### Design the comparison card

**Figure 2:**
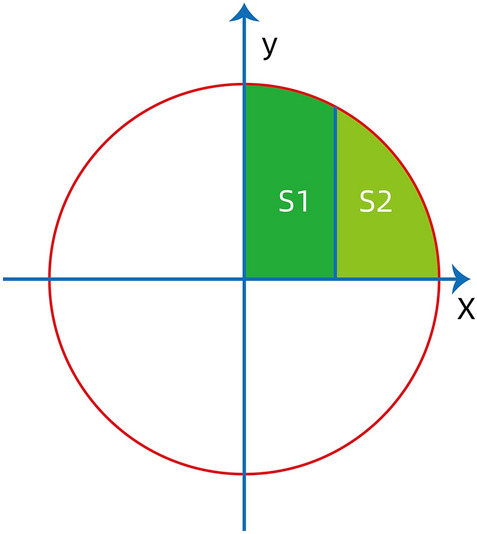
Illustration of the Riemann integral algorithm.

Taking a circle (radius = 4) as an example Fig. 2), the area of quarter circle was πr^2^/4 = 4π, then we calculated the coordinate of k value by calculus method to get two equal green area, as shown in Fig. 2,

$${\int }_{0}^{k}\surd 16 -{x}^{2}=2\pi $$

k = 1.615.

Therefore, a circle (radius = r) could be divided into n segments with equal area.

$${\int }_{0}^{k}\surd {r}^{2}-{x}^{2}=\pi {r}^{2}/4n$$

$${\int }_{0}^{k1}\surd {r}^{2}-{(x)}^{2}=\pi {r}^{2}/2n$$

**Figure 3:**
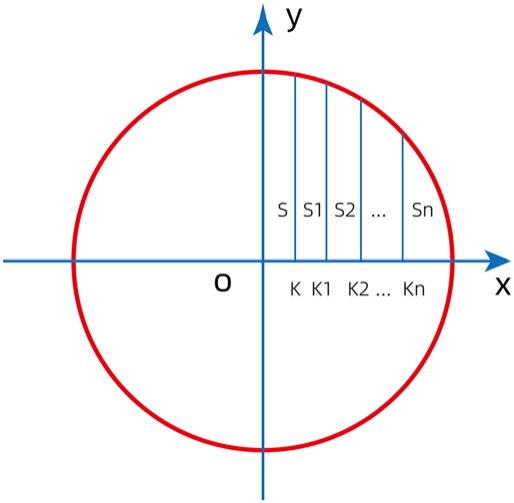
Find the cut point on the coordinate system using the Riemann integral algorithm.

Find k, k1…kn as shown in Fig. 3.

**Figure 4:**
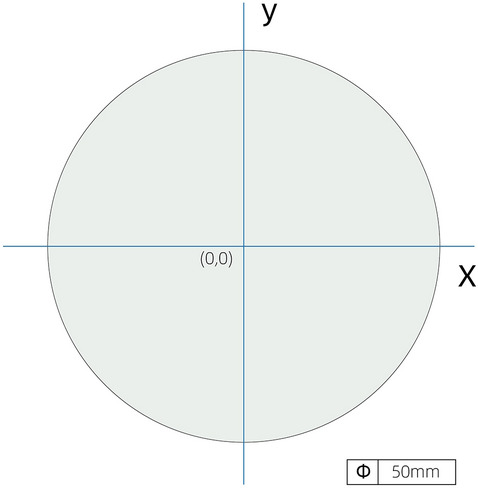
Establish the coordinate system on a circle with a diameter of 50 mm.

Drew a standard circle with a diameter of 50 mm in Geomagic Design Direct (USA Raindrop Company). Drew two vertical lines across the center as x-axis and y-axis, and the coordinate of center was (0,0) (Fig. 4).

Based on the requirement of single unit area as 2.5%, ten equal grids of a quarter circle should be obtained. Based on the calculus formula, n = 5, we got:

k = 3.94, k1 = 7.99, k2 = 12.29, k3 = 17.17.

The area of ten equal units was approximately 49 mm^2^, according to k*y = 49 mm^2^, k1*y1 = 49 mm^2^…

**Figure 5:**
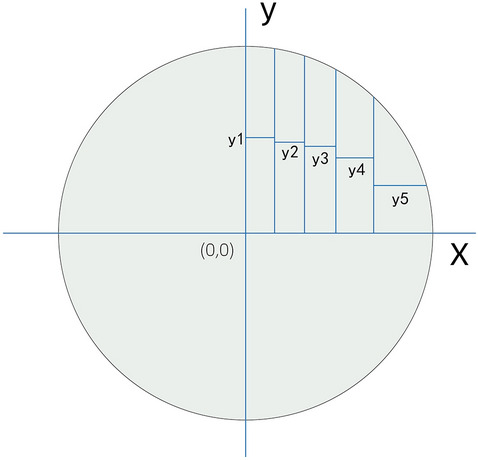
Find the corresponding coordinates dividing the 1/4 circle equally.

It could be calculated: y = 12.45, y1 = 12.13 y2 = 11.42 y3 = 10.06 y4 = 6.51 as shown in Fig. 5.

**Figure 6:**
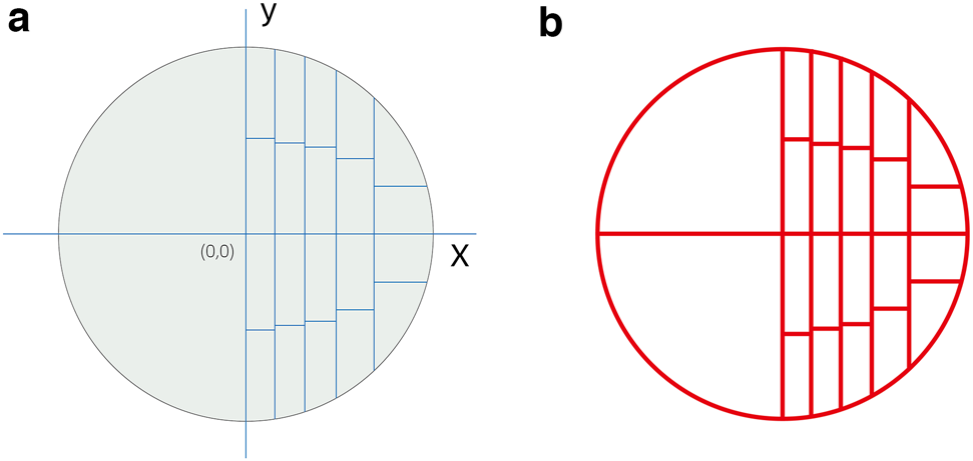
(**a**, **b**) Vectorized the comparison card.

Re-mirror the 1/4 circle to obtain a complete circle (Fig. 6a). We used Adobe Illustrator (Adobe company) to vectorize the picture and remove the background to obtain the comparison card with a high pixel resolution (Fig. 6b).

### Using of comparison card

**Figure 7:**
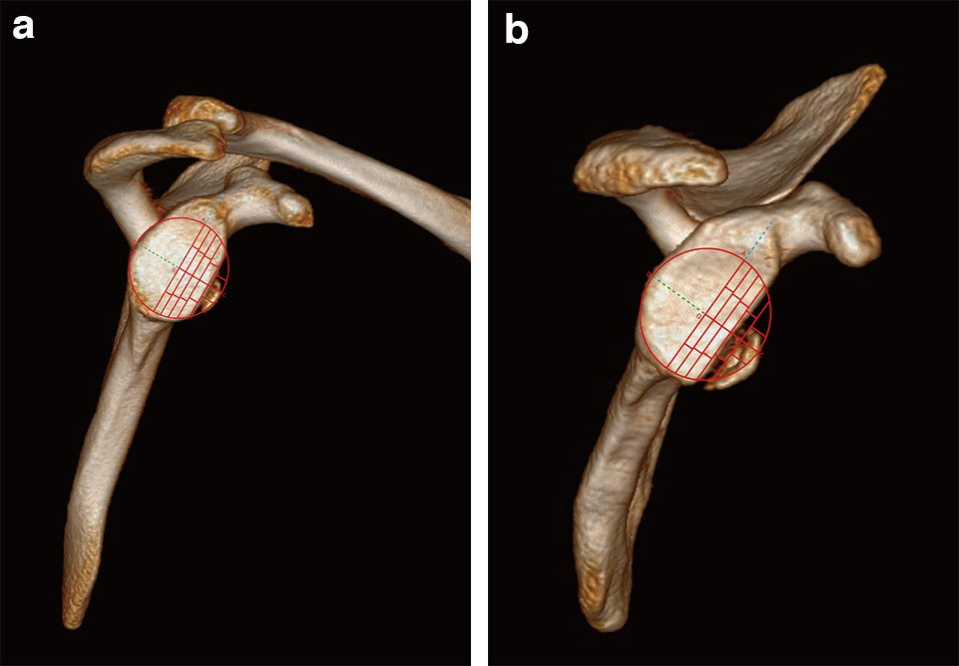
(**a**, **b**) Superimpose the comparison card with the en face view of glenoid.

The comparison card was transparent. Superimposed the card with the en face image of glenoid obtained from 3D CT scan (Fig. 7). We ensured that the center of comparison card and the best-fit circle of inferior glenoid coincided with each other. Then we zoomed the feature to correctly size the glenoid to match the card. As the area of each grid accounted for 2.5%, the defect percentage (p) equaled the number of grids (n) in the defect area multiple 2.5%: p = n*2.5%.

**Figure 8:**
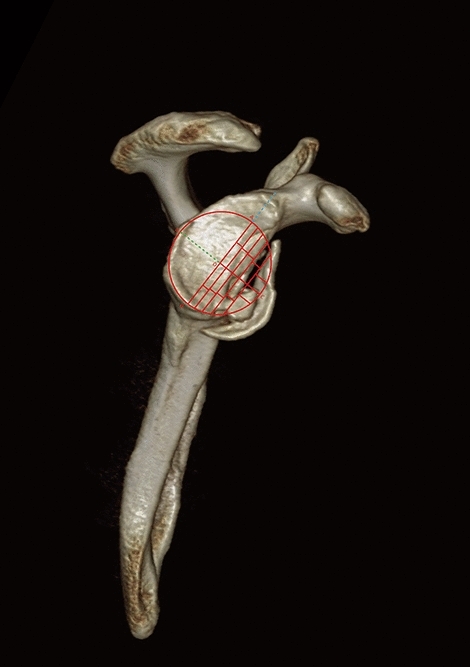
Estimation of measurement error.

If the bone defect could not fill the comparison card grid as shown in Fig. 8, it needed to be estimated by direct reading. To minimize the estimation error, the measurement accuracy could be improved by setting tolerance. Specifically defined as λ = 1.25% ± α, λ was the actual estimated value, α was the limit of tolerance. Through direct comparison and observation, if more than half of the grid was filled, it was calculated as 1.25% + α; if less than half of the grid, it was calculated as 1.25%-α; it was calculated as 1.25% when half of the grid was filled exactly. For statistical calculation, α was 0.5%. The limit error of a single grid was 0.75%, and the limit error of four grids was 3%, which was less than the current clinical requirement of 5%. Nevertheless, the accuracy requirement could be met.

### Sugaya method

**Figure 9:**
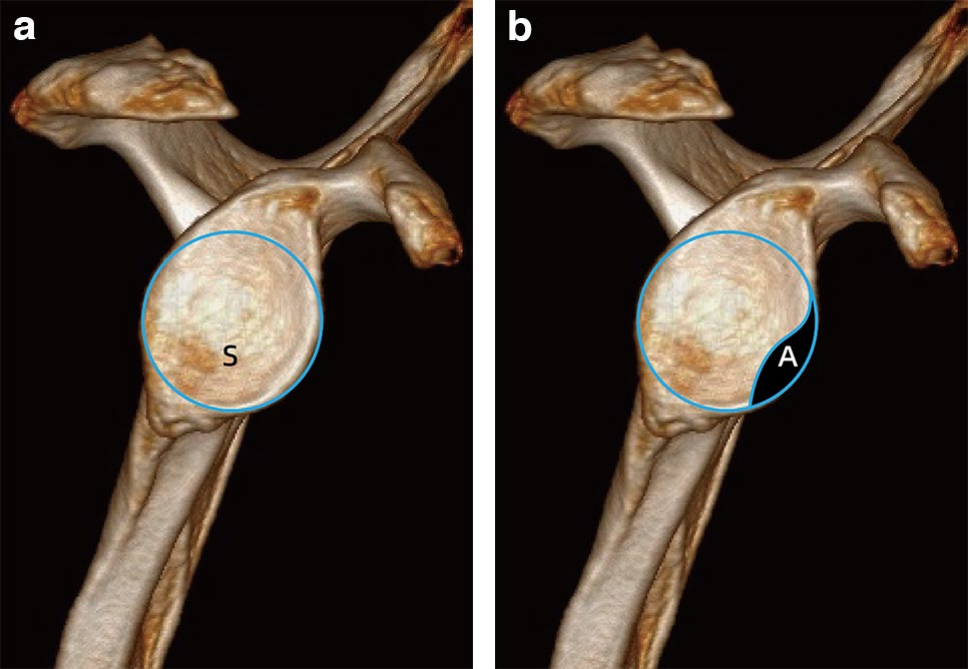
(**a**, **b**) Illustration of the Sugaya method.

Consideringthe inferior aspect of glenoid resembled a circle, we drew a circle that fit the outline of rim (Fig. 9), and calculated the circle area as S by Mimics 17.0. We draw along the defect contour using a free curve to completely divide the circle to obtain an irregular surface, and the area was A. The percentage of bone defect (D) was the ratio between area A and the area of assumed fitting circle (S), D = A/S. Bony defects could be divided into large (> 20%), medium (5–20%), and small (< 5%).

### Statistical analysis

The percentage of glenoid bone loss was represented as mean ± standard deviation. The comparison card and Sugaya method were used to measure the glenoid bone defect. Each measurement was performed on two separate occasions by a radiologist and two orthopedic surgeons, and there was 12 weeks’ interval between two measurements. The intra-group correlation coefficient (ICC) analysis was performed by three participants in 12 weeks, and the average value of inter-group correlation coefficient analysis was performed onthe two measurements by every participant. The reliability was considered good (ICC > 0.75), fair (0.4 < ICC < 0.75) or poor (ICC < 0.4)^18^. Statistical analysis was conducted using SPSS software version 25 (IBM, the U.S.). All tests were two-side, and *P* value less than 0.05 were considered statistically significant.

Agreement between the two methods was performed using Bland–Altman analysis. The average value of 3 observers was used as the final data of each glenoid. Statistical analysis was performed using MedCalc, version 15 (MedCalc Software Ltd).

## Data Availability

The datasets generated during and/or analysed during the current study are available from the corresponding author on reasonable request.
